# Supplementary figures and images for: Parsing social context in auditory forebrain of male zebra finches
Source: PLoS One. 2025 Mar 19;20(3):e0314795. doi: 10.1371/journal.pone.0314795 (PMC11922254; doi:10.1371/journal.pone.0314795)

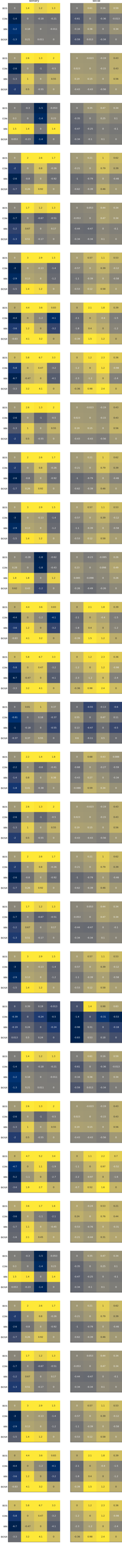

Supplement: S1 Data — Top: a second-long snippet of 9 out of 16 channels. Spikes are denoted with colored triangles; different colors denote different units. Bottom: a 400 ms subset of the snippet above. S2 Fig. Summary stimulus-triggered raster plot of all units for all stimuli. Each dot is a spike, each row is a stimulus presentation, and each block of rows is a unit. Rasters in red are from the solitary condition and rasters in blue are from the social condition. S1 Table. Stimulus presentations for each experiment. Number of stimulus presentations for each experiment. S1 File. Selectivity index values for each neuron for solitary and social audition. (ZIP) [file pone.0314795.s001.zip › supplement/S1 File.pdf]

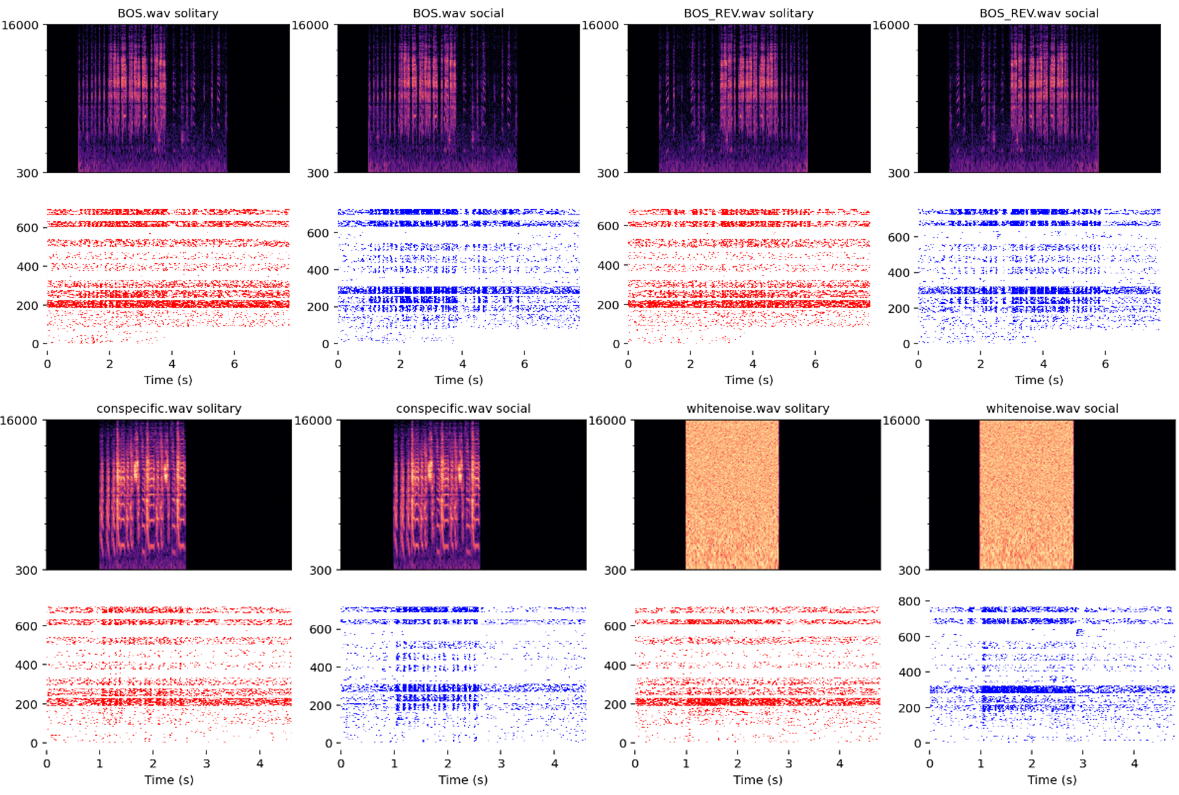

Supplement: S1 Data — Top: a second-long snippet of 9 out of 16 channels. Spikes are denoted with colored triangles; different colors denote different units. Bottom: a 400 ms subset of the snippet above. S2 Fig. Summary stimulus-triggered raster plot of all units for all stimuli. Each dot is a spike, each row is a stimulus presentation, and each block of rows is a unit. Rasters in red are from the solitary condition and rasters in blue are from the social condition. S1 Table. Stimulus presentations for each experiment. Number of stimulus presentations for each experiment. S1 File. Selectivity index values for each neuron for solitary and social audition. (ZIP) [file pone.0314795.s001.zip › supplement/S2 Fig.tif]
